# Preexisting ulcerative colitis increases the risk of immune-related colitis and predicts divergent survival outcomes in gastrointestinal cancer patients treated with immune checkpoint inhibitors

**DOI:** 10.3389/fimmu.2025.1627680

**Published:** 2025-08-13

**Authors:** Shuo Xu, Lu Chen, Kaixuan Liu, Hui Tao, Zhengzheng Ji, Jiamin Wu, Yuanyi Zhao, Qiankun Zhou, Liuying Li, Hanlong Zhu, Yunzhe Wang, Fangyu Wang

**Affiliations:** ^1^ Department of Gastroenterology and Hepatology, Jinling Hospital, Affiliated Hospital of Medical School, Nanjing University, Nanjing, Jiangsu, China; ^2^ Department of Gastroenterology and Hepatology, Cangzhou People’s Hospital, Cangzhou, Hebei, China; ^3^ Department of Rheumatology and Immunology, The Fourth Hospital of Hebei Medical University, Shijiazhuang, Hebei, China; ^4^ Department of Gastroenterology, Jinling Hospital, Nanjing Medical University, Nanjing, China; ^5^ Department of Hematology, The Fourth Hospital of Hebei Medical University, Shijiazhuang, Hebei, China

**Keywords:** immune checkpoint inhibitors, ulcerative colitis, immune-related colitis, gastrointestinal cancers, survival outcomes

## Abstract

**Background:**

The risk of immune-related colitis (IRC) and efficacy of immune checkpoint inhibitors (ICIs) in patients with gastrointestinal cancers and preexisting ulcerative colitis (UC) has not been well described.

**Patients and methods:**

We divided the patients with gastrointestinal cancers and preexisting UC who received ICIs between January 2021 and May 2024 into two groups as IRC group and non-IRC group. The electronic medical records were reviewed to compare the risk of IRC between two groups. Survival analysis and COX regression was conducted to assess clinical efficacy.

**Results:**

Of the 138 patients in study, 31 patients had a history of UC prior to initiation of immunotherapy. IRC occurred in 22 patients (71.0%) and over half experienced severe IRC (54.5%), a rate higher than that among similar patients without underlying UC (17.4%, *p* = 0.013). Compared with patients without UC who did not experience IRC, PFS and OS of patients with UC who had mild IRC were longer (PFS: 170 vs 96 days, *p* < 0.001; OS: 261 vs 172 days, *p* = 0.021) and those with severe IRC demonstrated merely a marginal advantage in terms of PFS (147 vs 96 days, *p* = 0.001), but no significant difference was observed in OS (171 vs 172 days, *p* = 0.851). The Multivariate analysis affirmed that mild IRC were correlated with a favorable prognosis (HR = 0.286, 95%CI: 0.106-0.769, *p* = 0.013), whereas severe IRC was not sufficient to be recognized as independent risk factors affecting survival outcomes. (HR = 1.149, 95%CI: 0.502-2.633, *p* = 0.742). The result of serum cytokines showed that the levels of IL-6 and IL-17A in patients with IRC were significantly elevated.

**Conclusion:**

For preexisting UC patients treated with ICIs, the risk of IRC is increased. Mild IRC may suggest a favorable prognosis, and being vigilant and effectively managing the occurrence of severe IRC is crucial for maximizing clinical benefits. Targeting the IL-6 pathway may be a potential new strategy for treating IRC in the future.

## Introduction

Gastrointestinal cancers have attracted the attention of researchers due to their high incidence and mortality rates (1). In the advanced stage, although the chemotherapy and radiotherapy have managed to extend the lifespan of patients, the effectiveness remains rather limited.

Immunotherapy with immune checkpoint inhibitors (ICIs) has changed the treatment paradigm in many tumor types and improved survival in a subset of patients with advanced or metastatic cancers (2, 3). The prominent members of this class of agents include the cytotoxic T lymphocyte-associated protein 4 (CTLA-4) inhibitor, the anti-programmed cell death 1 (PD-1) agents, and the anti-programmed cell death-ligand 1 (PD-L1) agents (4, 5). Despite their clinical efficacy, ICIs can induce various immune-related adverse events (irAEs) which limit their use in many patients. ICIs may affect peripheral tolerance to autoantigens, resulting in autoantibody formation, which could be associated with irAEs in various organs (6). ICIs can also activate T-cells with subsequent production of pro-inflammatory cytokines such as interferon-γ and tumor necrosis factor, which may result in excessive off-tumor inflammation and autoimmunity (7). The molecular and cellular mechanisms driving irAEs are poorly understood, as are the predisposing risk factors (8, 9). Out of concern that patients with underlying autoimmune diseases are at increased risk for developing severe irAEs, they have systematically been excluded from checkpoint inhibitor clinical trials (10).

Immune-related colitis (IRC), an intestinal toxic reaction mainly manifested by diarrhea and bloody stool, which is triggered by ICIs, is one of the most common adverse events associated with PD-1/PD-L1 inhibitor therapy (11, 12). IRC is a distinct clinical and pathologic entity but has many features resembling UC (13). The roles of PD-1/PD-L1 in UC are unclear. PD-1 and PD-L1 are expressed by the colonic epithelium, and surface expression of PD-1/PD-L1 is higher in UC patients, suggesting a potential regulatory function (14). Several meta-analyses have suggested retrospectively that ICIs are generally safe in patients with low active or untreated autoimmune diseases (15, 16). However, a multicenter retrospective study has indicated that patients with preexisting IBD who received immunotherapy have an increased risk of immune checkpoint inhibitors-related colitis (17). However, the generalizability of these conclusions is limited by constraints in patient numbers and disease heterogeneity. Given the heightened susceptibility of UC patients to various malignancies, some of which are indications for immunotherapy, a comprehensive understanding of the occurrence patterns and effect on treatment efficacy of IRC within the UC population is crucial (18, 19).

We present the characteristics and impact on outcomes of IRC in patients with gastrointestinal cancers and preexisting UC who received immunotherapy across multiple medical centers. To our knowledge, this is the first multicenter study to explore the relationship between IRC and prognosis in the preexisting UC population.

## Patients and methods

### Patient population

We performed a study in patients with preexisting UC (pre-UC) and gastrointestinal cancers, including esophageal cancer (EC), gastric cancer (GC), hepatocellular carcinoma (HCC) and colorectal cancer (CRC) with microsatellite instability-high (MSI-H), who received PD-1 inhibitors between January 2021 and May 2024 at the Fourth Hospital of Hebei Medical University, the Jinling Hospital of Nanjing University, and the Cangzhou People’s Hospital. Thereafter, a standardized data collection protocol was used among all centers to ensure consistency in the variables collected. Patients were included only if they had clear documentation of UC. Eligible patients were identified through searches of institutional databases and electronic medical records. To compare the characteristics of IRC, we included a control cohort of patients without UC (non-UC) who received the same therapy and documented information of them. Determination of presence and grade of IRC in the control group was done in a similar fashion to that of patients with UC.

### Clinical characteristics

The clinical information we collected from electronic medical records were as follows: age, gender, smoking history, Eastern Cooperative Oncology Group performance status (ECOG PS). Variables related to oncologic history included cancer type, disease status, treatment line, treatment therapy, immune checkpoint inhibitor type and treatment duration.

### IRC diagnosis and assessment

The inflammatory side effects of the colon characterized by diarrhea and bloody stool due to immune tolerance imbalance induced by ICIs were defined as IRC. The information of diarrhea and bloody stools (or fecal occult blood) was documented, and peak grade of IRC according to the Common Terminology Criteria for Adverse Events version 5.0. IRC was evaluated by at least 2 or more clinical senior oncologists. Adverse events classified as grade 3–5 are considered severe IRC.

Given that there is currently no recognized gold standard for differentiating UC from IRC, we have developed a practical set of diagnostic criteria based on current clinical practice and relevant guidelines. Patients with the following characteristics are more likely to be diagnosed with IRC: (1) New gastrointestinal symptoms occur after the initiation of ICIs, or their clinical manifestations do not conform to the typical acute flare-up features of UC in the patient’s past; (2) Endoscopic examination shows a lesion distribution different from that of UC, and the histopathology is significantly distinct from the typical pathological changes of UC (such as obvious intraepithelial lymphocyte infiltration, cryptitis or crypt abscess, etc.). Considering that some UC patients may have incompletely healed mucosa before receiving immunotherapy, which may interfere with the endoscopic assessment of IRC, this study excluded UC cases with moderate to severe endoscopic abnormalities and only included patients with normal or mild endoscopic findings to enhance the reliability of endoscopic diagnosis of IRC; (3) Show rapid and significant clinical remission to glucocorticoid treatment (especially intravenous administration), or symptoms improve rapidly after the suspension of ICIs treatment; (4) Infectious causes have been excluded through systematic screening. The final diagnosis was independently evaluated by at least two clinical experts with senior professional titles (only referring to information related to IRC) to ensure the objectivity and consistency of the diagnostic conclusion.

### UC information

We recorded the duration between the diagnosis of UC or the most recent active episode of UC and the commencement of immunotherapy. Previous treatments for UC patients were categorized as mesalamine or immunosuppressants. The findings from the latest available endoscopic assessment were documented and classified as normal or mild. Furthermore, we documented the presence of extraintestinal manifestations related to UC.

### Cancer treatment and assessment

Patients received standard anti-PD-1 antibody every 21 days until disease progression, clinical deterioration, unacceptable toxicity, or patient’s refusal. Following the initiation of treatment, clinical and laboratory tests were carried out as clinically indicated each cycle before drug administration. Body computed tomography (CT) scans were taken every 2–3 cycles. Objective tumor response was evaluated according to the Response Evaluation Criteria in Solid Tumors (RECIST) version 1.1.

### Serum samples and cytokine assay

At the time of IRC occurrence, blood samples were collected from patients via venipuncture, and control samples were simultaneously collected from patients without irAEs. All samples were centrifuged at 1,000 × g for 10 minutes at 4°C, and the serum was then separated and aliquoted. Sub-samples were stored at -80°C. The levels of cytokines in the serum were quantitatively analyzed using a cytokine multiplex detection platform and enzyme-linked immunosorbent assay (ELISA).

### Statistical analysis

A descriptive summary of continuous variables using medians and interquartile ranges (IQRs) while categorical variables using frequencies and percentages was performed. Fisher’s exact test was used to compare categorical variables, and the Wilcoxon rank-sum test was used to compare continuous variables. Progression-free survival (PFS) was calculated from the date of the first dose to the date of progression, death, or last follow-up, whichever came first. The overall survival (OS) data were calculated from the diagnosis until death or censored at the last date of the follow-up. To determine the association between the incidence of IRC and prognosis, objective response rate (ORR) and disease control rate (DCR) were performed. The association between the IRC and prognosis was analyzed using the Kaplan-Meier method. A Cox proportional hazards regression model was constructed, and the possible factors influencing the prognosis of patients (including baseline clinical characteristics such as age, gender, ECOG PS score, and the occurrence of IRC) were included in univariate and multivariate analyses to explore whether IRC is an independent risk factor for prognosis.

For the analyses described above, the following software programs were utilized: SPSS Statistics version 26.0 (IBM Corporation) for Fisher’s exact test, the Wilcoxon rank-sum test and Cox regression analysis, GraphPad Prism version 9.0 (San Diego, CA, USA) for Kaplan-Meier analysis. All *p* values were two-sided, and *p* < 0.05 was considered to indicate a statistically significant difference.

## Results

### Patients

A total of 107 patients in non-UC group and 31 in pre-UC group were involved in this study. Our focus was specifically on patients with EC, GC, HCC and CRC (MSI-H) at stage III and IV, among whom 70.3% were male and 29.7% were female. The clinical characteristics were listed in **Table 1** and the ICIs types of patients were showed in **Supplementary Table S1**. No significant differences were observed between the two groups in terms of gender, age, smoking, ECOG PS, cancer type, cancer stage, treatment line and treatment therapy. However, patients in pre-UC group exhibited a higher incidence of IRC (71.0% vs 21.5%, *p* < 0.001).

**Table 1:** Patient characteristics.

IRC	Total no. (%)	non-UC no. (%)	pre-UC no. (%)	*p* value
Total N	138 (100)	107 (77.5)	31 (22.5)	
Gender				
male	97 (70.3)	76 (71.0)	21 (67.7)	0.824
female	41 (29.7)	31 (29.0)	10 (32.3)	
Age				
<65	52 (37.7)	45 (42.1)	7 (22.6)	0.059
≥65	89 (62.3)	62 (57.9)	24 (77.4)	
Smoking				
no	52 (37.7)	39 (36.4)	13 (41.9)	0.675
yes	86 (62.3)	68 (63.6)	18 (58.1)	
ECOG PS				
0	80 (58.0)	64 (59.8)	16 (51.6)	0.536
1-2	58 (42.0)	43 (40.2)	15 (48.4)	
Cancer type				
ESCC	24 (17.4)	20 (18.7)	4 (12.9)	0.910
GC	70 (50.7)	53 (49.5)	17 (54.8)	
HCC	40 (29.0)	31 (29.0)	9 (29.1)	
CRC (MSI-H)	4 (2.9)	3 (2.8)	1 (3.2)	
Cancer stage				
III	91 (65.9)	71 (66.4)	20 (64.5)	1.000
IV	47 (34.1)	36 (33.6)	11 (35.5)	
Treatment line				
≤2	68 (49.3)	54 (50.5)	14 (45.2)	0.685
≥3	70 (50.7)	53 (49.5)	17 (54.8)	
Combined with chemotherapy/targeted therapy				
no	42 (30.4)	32 (29.9)	10 (32.3)	0.827
yes	96 (69.6)	75 (70.1)	21 (667.7)	
IRC				
no	93 (67.4)	84 (78.5)	9 (29.0)	< 0.001
yes	45 (32.6)	23 (21.5)	22 (71.0)	

ECOG PS, Eastern Cooperative Oncology Group performance status; EC, esophageal cancer; GC, gastric cancer; HCC, hepatocellular carcinoma; CRC, colorectal cancer; IRC, immune-related colitis.

In the pre-UC group, clinical information of UC was also gathered. As shown in **Table 2**, the median duration from UC diagnosis to commencement of immunotherapy was 16 years, and the median interval from the last UC episode to immunotherapy was 5 years. The majority of people were given mesalamine only to treat UC before receiving immunotherapy (further details on immunosuppressive treatments was listed in **Supplementary Table S2**). Furthermore, more than 90% of patients did not receive any treatment for UC when they started ICIs therapy. 74.1% of the patients exhibited no significant endoscopic lesions at the onset of immunotherapy, and no patients had complications and extraintestinal manifestations before the commencement of immunotherapy.

**Table 2:** Baseline UC data.

Characteristic	No. of patients (%, n = 31)
Median time from UC diagnosis to immunotherapy, years (IQR)	16 (8-24)
Median time from last UC episode to immunotherapy, years (IQR)	5 (4-8)
UC treatment before immunotherapy	
mesalamine	23 (74.1)
immunosuppressive	8 (25.9)
UC treatment at time of immunotherapy initiation	
no	29 (93.5)
yes	2 (6.5)
Severity of endoscopic findings of UC before immunotherapy	
normal	23 (74.1)
mild	8 (25.9)
Extraintestinal manifestation before immunotherapy	
no	31 (100)
yes	–
Complications from UC	
no	31 (100)
yes	–
Surgery for UC	
no	31 (100)
yes	–

Complications of UC include colonic stricture, perforation; Extraintestinal manifestation of UC consisted of arthritis, blood clot, nephrolithiasis, and primary sclerosing cholangitis; Immunosuppressive therapy includes corticosteroid, azathioprine, and mercaptopurine.

### Toxicity

In **Table 3**, we outlined the characteristics of IRC. Compared to the non-UC group, the pre-UC group had a significantly higher probability of developing severe IRC (54.5% vs 17.4%, *p* = 0.013), a shorter median time from the start of immunotherapy to onset of IRC (46 vs 58 days, *p* = 0.011), a longer median duration of IRC (20 vs 13 days, *p* = 0.005). Unlike the majority of non-UC patients who merely needed observation or symptomatic treatment to reduce IRC, a higher proportion of pre-UC patients required corticosteroid to control their symptoms. Additionally, a greater proportion of pre-UC patients discontinued immunotherapy because of IRC (54.5% vs 17.4%, *p* = 0.013) and experienced relapse after IRC remission (36.4% vs 8.7%, *p* = 0.035). These findings suggest that patients with preexisting UC are at an increased risk for developing IRC following immunotherapy.

**Table 3:** Characteristics of IRC.

Stop immunotherapy due to IRC	non-UC no. (%, n = 23)	pre-UC no. (%, n = 22)	*p* value
Grade			
1	10 (43.5)	3 (13.6)	0.022
2	9 (39.1)	7 (31.8)	
3	4 (17.4)	8 (36.4)	
4	0 (0.0)	4 (18.2)	
Severity of IRC			
mild	19 (82.6)	10 (45.5)	0.013
severe	4 (17.4)	12 (54.5)	
Median time from immunotherapy to IRC, days (IQR)			
	58 (46, 69)	46 (41, 54)	0.011
Median duration of symptoms, days (IQR)			
	13 (9, 17)	20 (16, 25)	0.005
Treatment of IRC			
no	10 (43.5)	3 (13.6)	0.021
symptomatic treatment	9 (39.1)	7 (31.8)	
corticosteroid	4 (17.4)	12 (54.5)	
Subsequent recurrent IRC			
no	21 (91.3)	14 (63.6)	0.035
yes	2 (8.7)	8 (36.4)	
Median time from treatment to remission, days (IQR)			
	102 (99, -)	68 (53, 78)	0.444
Stop immunotherapy due to IRC			
no	19 (82.6)	10 (45.5)	0.013
yes	4 (17.4)	12 (54.5)	
Mortality as a result of IRC			
	0 (0.0)	0 (0.0)	1.000

Severity of IRC (mild), grade 1-2; Severity of IRC (severe), grade 3-5; IQR, interquartile range.

### Response to immunotherapy

**Figure 1:**
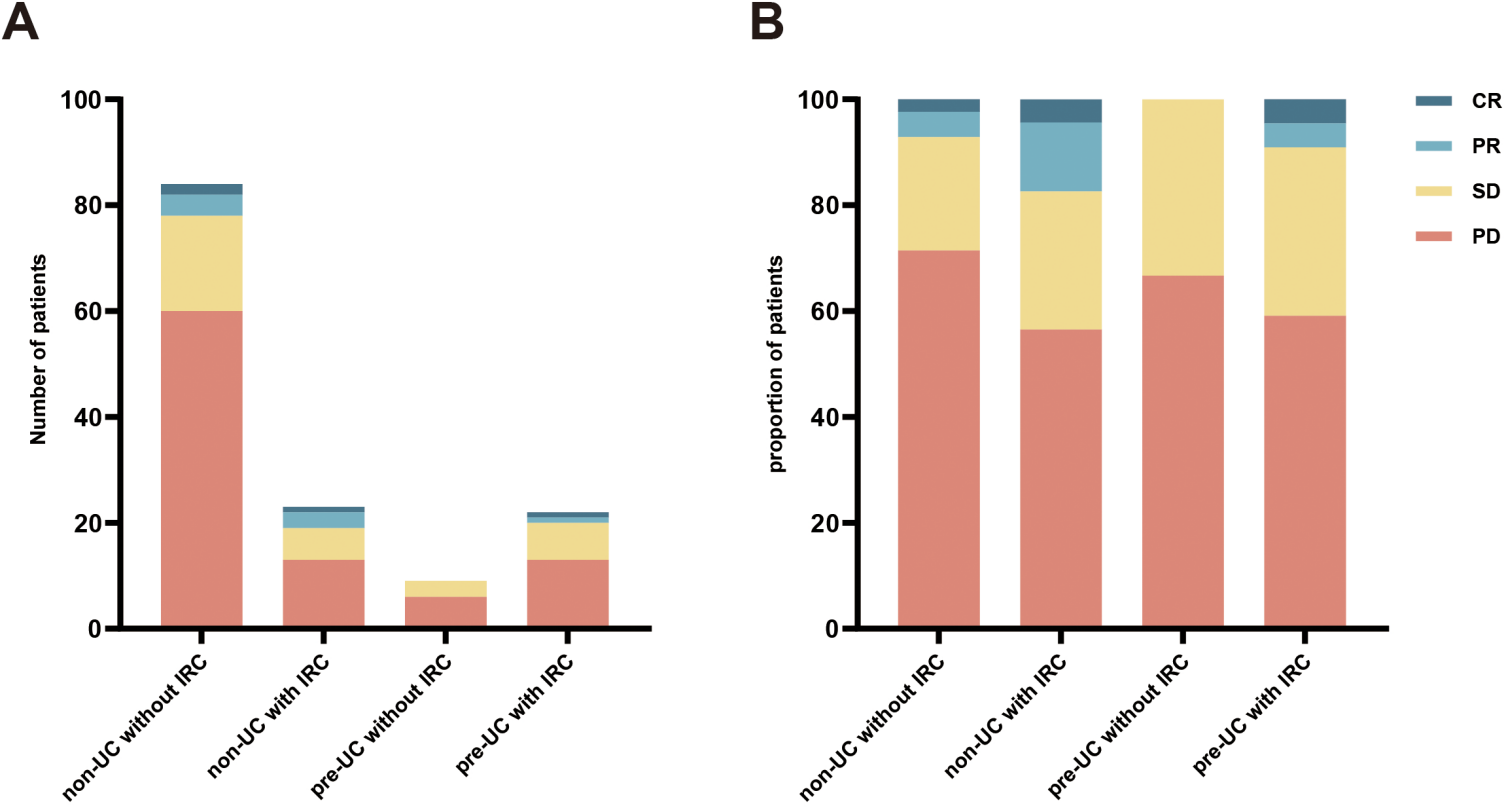
Response to immunotherapy. **(A)** the number of different patients in the four groups; **(B)** the proportion of different patients in the four groups.

Data on tumor dynamics following immunotherapy was gathered from all patients (**Figure 1**) and DCR and ORR were calculated and presented in **Table 4**. Compared with the patients without IRC in non-UC group, we observed higher ORR and DCR in patients with IRC of non-UC group (ORR: 17.4% vs 7.1%, *p* = 0.217; DCR: 43.5% vs 28.6%, *p* = .0174). A slight advantage in DCR was observed for patients in the pre-UC group without IRC (DCR: 33.3% vs 28.6%, *p* = .0716). In pre-UC patients with IRC, despite the superiority of DCR (DCR: 40.9% vs 28.6%, *p* = 0.266), the increase in ORR is not significant (ORR: 9.1% vs 7.1%, *p* = 0.670). Unfortunately, we did not observe a statistical difference in DCR or ORR mentioned above due to the insufficiency of the sample size.

**Table 4:** Response to immunotherapy.

Group	DCR (%)	*P* value	ORR (%)	*P* value
non-UC without IRC	28.6 (18.7-38.4)	Reference	7.1 (1.5-12.8)	Reference
non-UC with IRC	43.5 (21.6-65.4)	0.174	17.4 (0.6-34.2)	0.217
pre-UC without IRC	33.3 (0.0-71.8)	0.716	–	–
pre-UC with IRC	40.9 (18.6-63.2)	0.266	9.1 (0.0-22.1)	0.670

### Cancer outcomes

**Figure 2:**
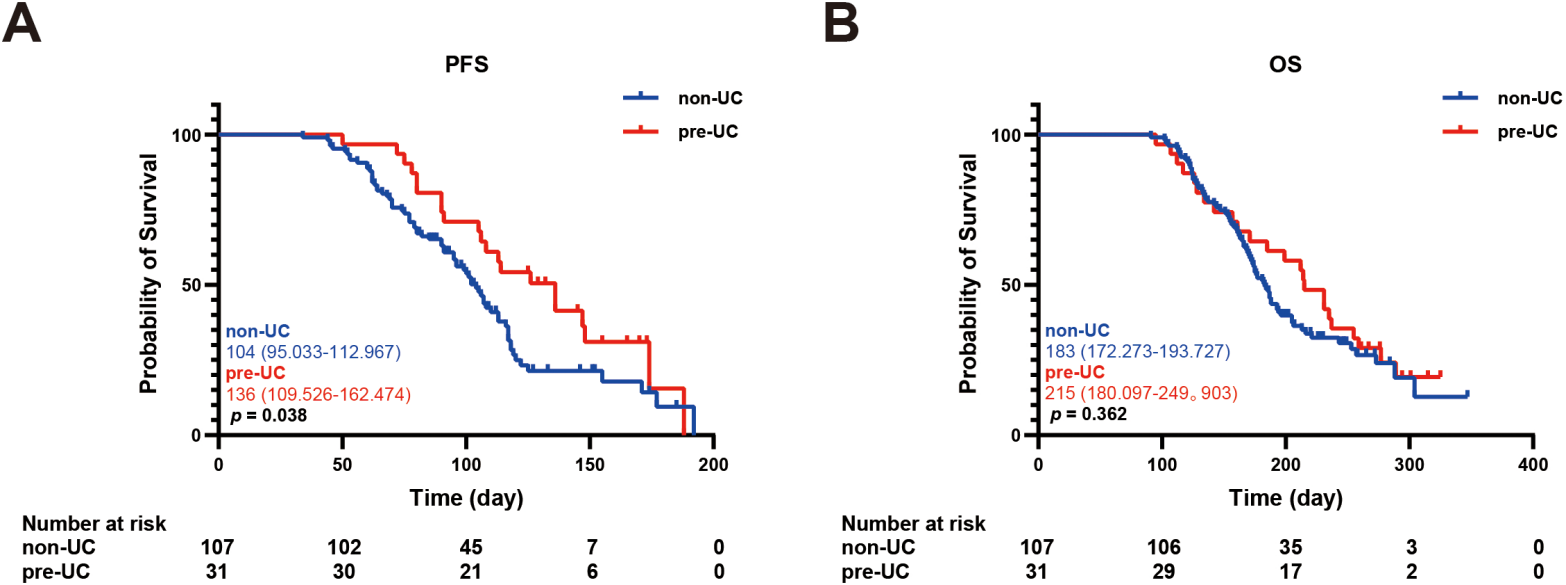
Kaplan-meier survival curve of PFS and OS. **(A)** The Kaplan-Meier curve of PFS (non-UC vs pre-UC). **(B)** The Kaplan-Meier curve of OS (non-UC vs pre-UC).

**Figure 3:**
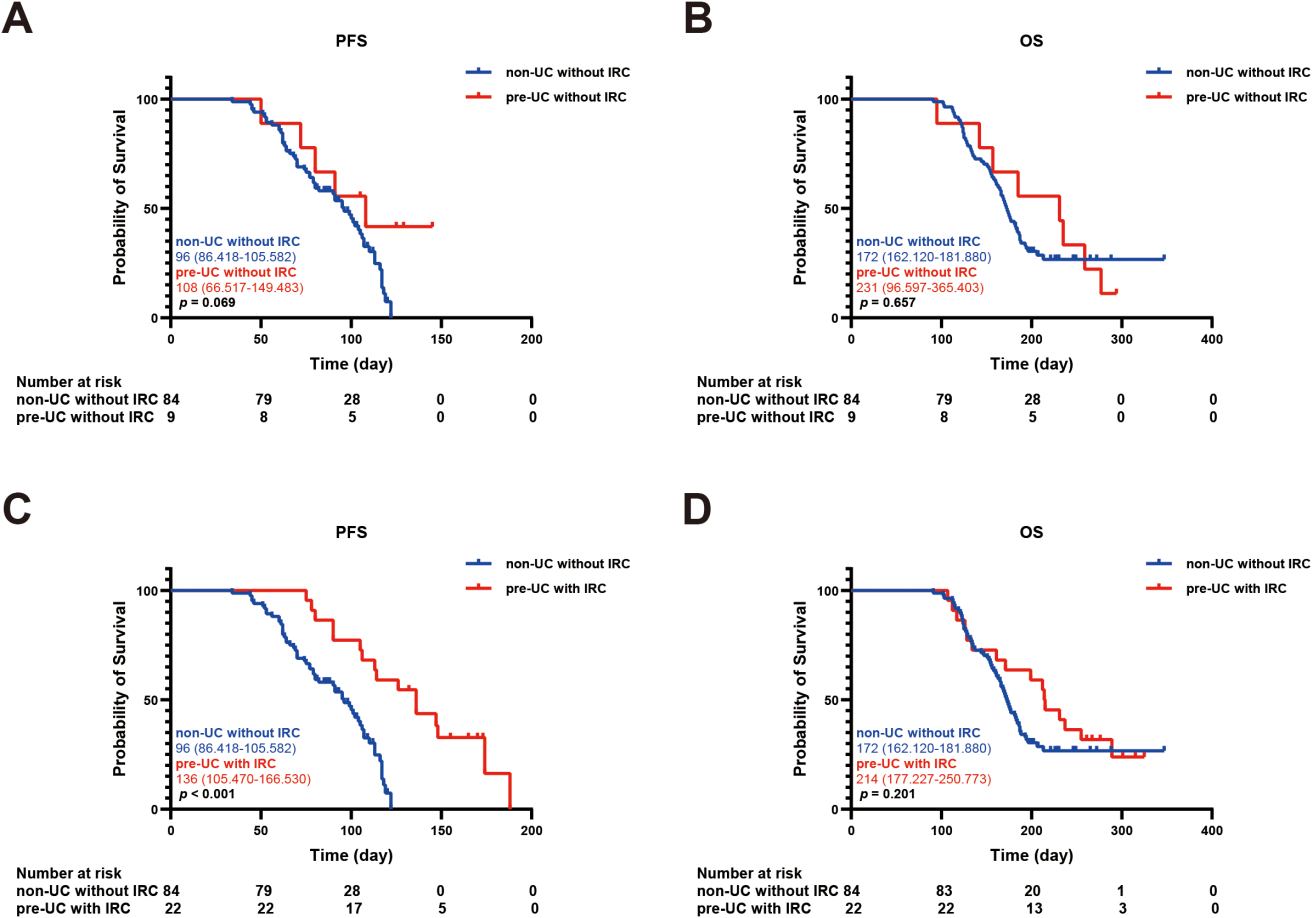
Kaplan-meier survival curve of PFS and OS. **(A)** The Kaplan-Meier curve of PFS (non-UC without IRC vs pre-UC without IRC). **(B)** The Kaplan-Meier curve of OS (non-UC without IRC vs pre-UC without IRC). **(C)** The Kaplan-Meier curve of PFS (non-UC without IRC vs pre-UC with IRC). **(D)** The Kaplan-Meier curve of OS (non-UC without IRC vs pre-UC with IRC).

**Figure 4:**
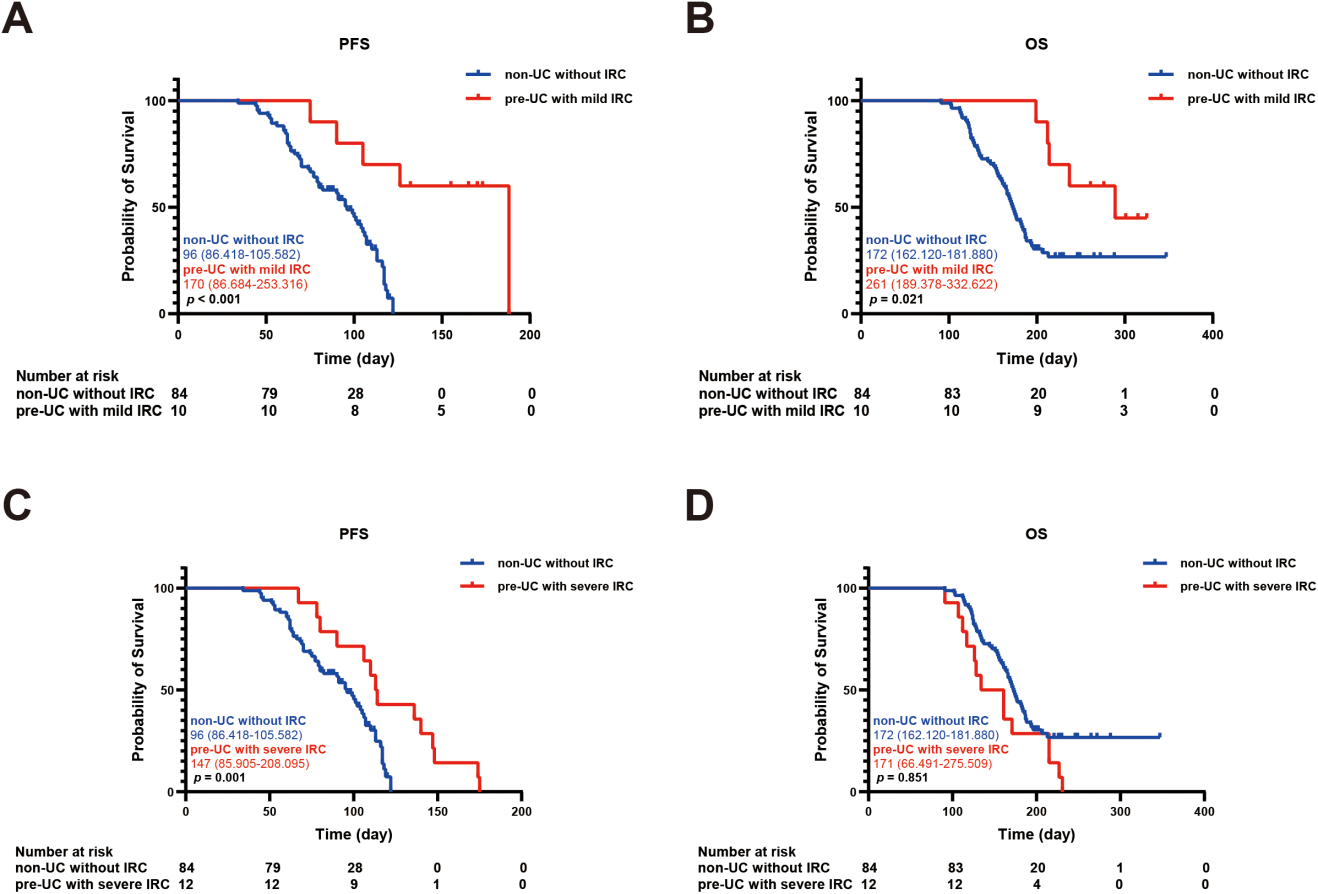
Kaplan-meier survival curve of PFS and OS. **(A)** The Kaplan-Meier curve of PFS (non-UC without IRC vs pre-UC with mild IRC). **(B)** The Kaplan-Meier curve of OS (non-UC without IRC vs pre-UC with mild IRC). **(C)** The Kaplan-Meier curve of PFS (non-UC without IRC vs pre-UC with severe IRC). **(D)** The Kaplan-Meier curve of OS (non-UC without IRC vs pre-UC with severe IRC).

In order to explore the relationship between IRC and prognosis, we calculated and analyzed the PFS and OS. We found that the PFS in the pre-UC group was longer than that in the non-UC group, but there was no notable disparity in OS (PFS: 136 vs 104 days, *p* = 0.038; OS: 215 vs 183 days, *p* = 0.362) (**Figures 2A, B**).Using the survival outcomes of non-UC patients without IRC as a reference, our analysis indicated that pre-UC patients without IRC demonstrated an advantage in both PFS and OS, although these differences were not statistically significant (PFS: 108 vs 96 days, *p* = 0.069; OS: 231 vs 172 days, *p* = 0.657) (**Figures 3A, B**). A similar trend was observed in PFS but not OS of pre-UC patients with IRC (PFS: 136 vs 96 days, *p* < 0.001; OS: 214 vs 172 days, *p* = 0.201) (**Figures 3C, D**). To further explore the reasons for the discrepancies in PFS and OS, we categorized IRC into mild (grade 1-2) and severe (grade 3-5), and compared these groups separately to non-UC patients without IRC. The results showed that PFS and OS in pre-UC patients with mild IRC were significantly improved (PFS: 170 vs 96 days, *p* < 0.001; OS: 261 vs 172 days, *p* = 0.021) (**Figures 4A, B**). However, pre-UC patients with severe IRC showed an advantage in PFS (PFS: 147 vs 96 days, *p* = 0.001), but no significant difference was observed in OS compared with non-UC patients without IRC (OS: 171 vs 172 days, *p* = 0.851) (**Figures 4C, D**).

Additionally, we incorporated gender, age, smoking history, ECOG PS, cancer type, cancer stage, number of treatment lines, treatment regimen and IRC status into the COX model. Both univariate and multivariate analyses were conducted to assess the relationship between IRC and OS (**Table 5**). The analysis results indicated that none of the baseline characteristics (including gender, age, smoking history, ECOG PS, cancer type, cancer stage, number of treatment lines and treatment regimen) were independent risk factors affecting OS, thus ruling out their influence on prognosis. Univariate analysis revealed that pre-UC patients with mild IRC had a decreased risk of mortality (HR = 0.346, 95%CI: 0.145-0.823, *p* = 0.016). Further multivariate analysis also confirmed that mild IRC was associated with a more favorable prognosis (HR = 0.286, 95%CI: 0.106-0.769, *p* = 0.013), but in the pre-UC population, severe IRC was not sufficient to be recognized as independent risk factors affecting survival outcomes. (HR = 1.149, 95%CI: 0.502-2.633, *p* = 0.742).

**Table 5:** Univariate and multivariate analyses of OS with Cox regression models.

Group	Univariate analysis				Multivariate analysis			
	HR	95% CI		*P* value	HR	95% CI		*P* value
		Lower	Upper			Lower	Upper	
Gender								
male	Reference				Reference			
female	0.650	0.434	0.972	0.036	0.628	0.342	1.152	0.133
Age								
<65	Reference				Reference			
≥65	1.086	0.735	1.605	0.678	1.164	0.754	1.797	0.492
Smoking								
no	Reference				Reference			
yes	1.308	0.886	1.933	0.177	1.058	0.592	1.892	0.849
ECOG PS								
0	Reference				Reference			
1-2	1.089	0.738	1.605	0.668	1.137	0.674	1.917	0.630
Cancer type								
ESCC	Reference				Reference			
GC	0.582	0.338	1.002	0.051	0.739	0.408	1.339	0.319
HCC	0.938	0.532	1.653	0.824	1.353	0.644	2.841	0.425
CRC (MSI-H)	0.418	0.097	1.805	0.243	1.111	0.232	5.328	0.895
Cancer stage								
III	Reference				Reference			
IV	1.213	0.803	1.833	0.359	1.050	0.650	1.695	0.843
Treatment line								
≤2	Reference				Reference			
≥3	1.476	1.030	1.859	0.081	1.294	0.892	1.517	0.126
Combined with chemotherapy/targeted therapy								
no	Reference				Reference			
yes	0.685	0.446	1.053	0.084	0.581	0.361	1.035	0.055
Gastrointestinal toxicity in different groups								
non-UC without IRC	Reference				Reference			
pre-UC without IRC	0.781	0.368	1.656	0.519	0.596	0.250	1.419	0.242
pre-UC with IRC	0.722	0.436	1.197	0.206	0.459	0.195	1.080	0.074
pre-UC with mild IRC	0.346	0.145	0.823	0.016	0.286	0.106	0.769	0.013
pre-UC with severe IRC	0.927	0.422	2.037	0.851	1.149	0.502	2.633	0.742

### IRC-related cytokines

**Figure 5:**
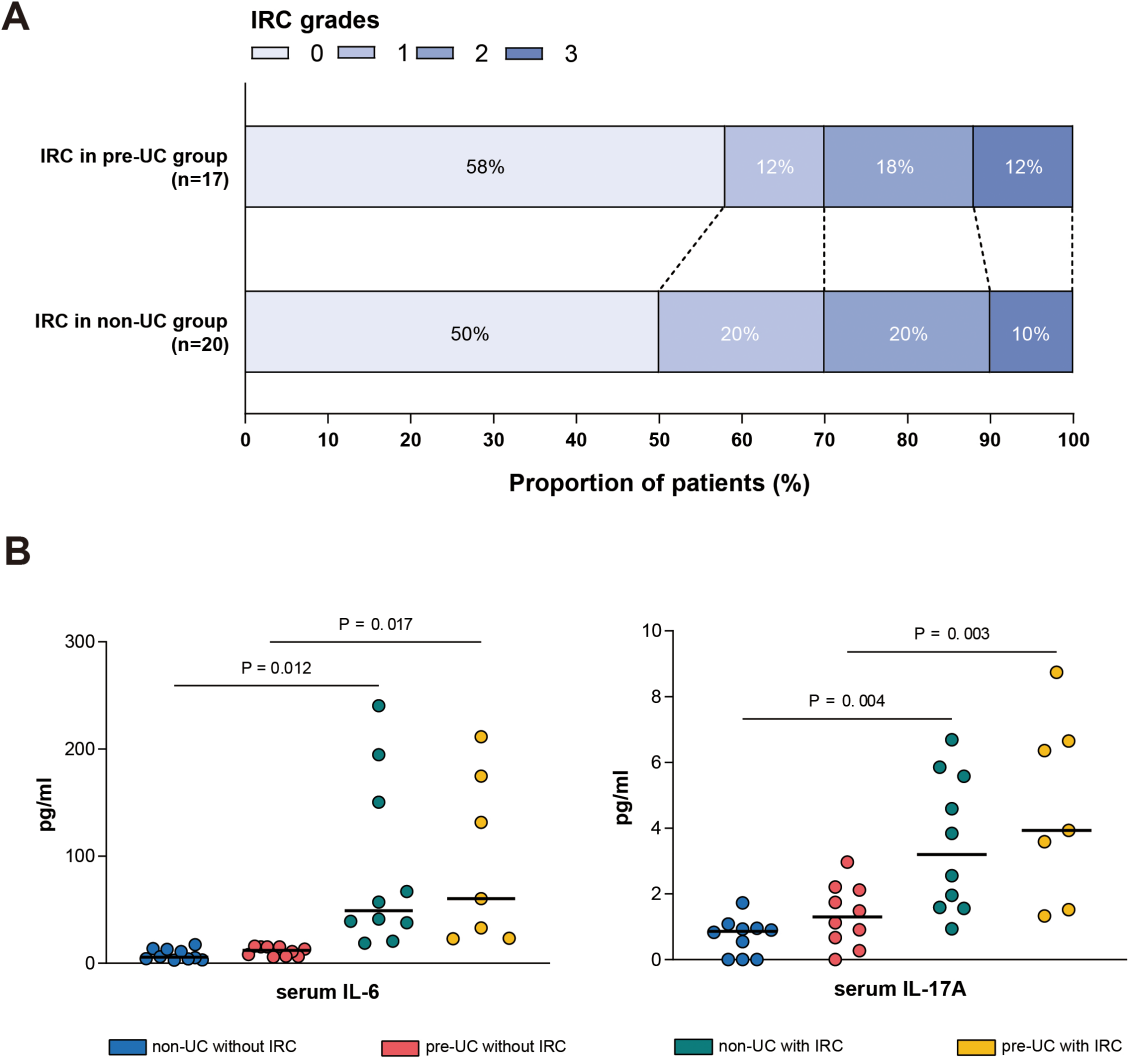
Serum cytokine levels in different groups. **(A)** Distribution of IRC grades (Grade 0-3) among patients in the pre-UC group (n = 17) and non-UC group (n = 20). **(B)** Serum concentrations of IL-6 (left) and IL-17A (right) measured in different patients.

We collected peripheral blood samples from some patients with IRC and detected the levels of IL-6 and IL-17A in their serum, comparing them with the cytokine levels of patients without IRC during the same period. The results showed that in both the non-UC group and the pre-UC group, the levels of IL-6 and IL-17A in the serum of patients with IRC were significantly elevated (**Figure 5**).

## Discussion

This is the largest multicenter study to date examining the risk of IRC and its relationship with clinical prognosis in gastrointestinal cancer patients with preexisting UC who received anti-PD-1 antibodies. The result indicate significantly higher incidence of IRC in preexisting UC patients compared to those without UC. Notably, higher rates of severe IRC was observed in pre-UC group, along with an increased frequency of symptom recurrence post-treatment. However, there were no fatalities directly attributable to IRC recorded.

Our findings diverge from previous reports of immunotherapy in patients with underlying UC. Previous analyses of patients with underlying autoimmune diseases only included a limited number of UC patients and offered scant data on UC histories and prior diagnostic evaluations (20, 21). A multicenter retrospective study has previously explored the risk of IRC in IBD, but it did not further examine the relationship between IRC and prognosis (17). We have provided substantially more clinical details of UC than those previously reported, thereby establishing a clearer relationship between IRC and prognosis in UC, reducing the impact of selection bias and the variability due to disease heterogeneity, and facilitating the application of our results to other clinical situations.

In Our study, people without a history of UC showed that IRC were connected with longer PFS or OS (**Supplementary Figures S1A, B**), in line with previous studies (6). Patients in the pre-UC group without IRC also exhibited improved PFS and OS compared to those without IRC in non-UC group, although statistical significance was not achieved. We hypothesized that this effect may be attributed to the relatively active immune system in pre-UC patients, potentially leading to a higher response rate following anti-PD-1 antibody treatment. This hypothesis was supported in pre-UC patients with mild IRC, as their PFS and OS prolongation was more pronounced. However, it is important to note that this advantage of OS did not increase with the aggravation of IRC in pre-UC patients. This might imply that pre-UC patients with severe IRC could benefit less from immunotherapy, even though they showed advantages in PFS. This seemingly contradictory phenomenon can be explained from the following two aspects: Firstly, the clinical management of severe IRC usually includes the use of corticosteroids or other immunosuppressants, and ICIs may be suspended or even permanently discontinued. Such intervention may lead to the suppression of the immune system’s anti-tumor function, thereby affecting the patient’s long-term survival. Secondly, severe IRC often presents with severe symptoms such as diarrhea and bloody stools, which may lead to a series of systemic complications such as electrolyte imbalance and anemia. These non-tumor-related complications may directly increase the risk of death for the patient.

Previous meta-analyses have demonstrated a positive association between IRC and favorable outcomes. While the incidence of IRC was higher in IBD, the increased response rate of immunotherapy suggested that the benefits in this population likely outweighed the risks (22). Nevertheless, our results indicated that this conclusion mentioned above may not be universally applicable to all patient types. The relatively active immune system observed in pre-UC patients may signify more effective tumor-killing effect. However, the presence of IRC could indicate more intense autoimmune damage, particularly in pre-UC patients with severe IRC. This delicate balance between antitumor effect and autoimmune damage should be carefully considered when using ICIs in pre-UC population. Furthermore, larger studies with substantial sample sizes are needed to assess the rationality of immunotherapy in patients with underlying UC (23).

In the pre-UC cohort, a considerable proportion of patients developed severe IRC and received corticosteroid treatment. Currently, there is still considerable controversy over whether corticosteroids affect the anti-tumor efficacy of ICIs. Although it is theoretically speculated that corticosteroids may inhibit T-cell function through multiple mechanisms and thereby interfere with the anti-tumor effect of ICIs, only a few studies support this hypothesis. Notably, the corticosteroid treatment regimens used in these studies were mostly high-dose and long-term, and they all had certain limitations. For instance, when patients received corticosteroids to treat irAEs, they often discontinued ICIs treatment at the same time. Therefore, it is still unclear whether the shortened survival period is due to the use of corticosteroids or the interruption of ICIs treatment. A study in 2019 indicated that the use of high-dose corticosteroids in combination with other anti-tumor treatments at baseline might have an adverse effect on patients’ survival time, while low-dose application or corticosteroids not used due to the tumor itself did not show a significant impact on patients’ prognosis (24). The NCCN guidelines also point out that the use of corticosteroids to treat irAEs does not negatively affect the treatment outcome of tumors, a view consistent with the majority of research results. In this study, for patients with severe IRC, the corticosteroid treatment adopted was all low-dose, and the dosage was gradually reduced and stopped in a timely manner after symptom relief, thereby minimizing the potential impact of corticosteroids. Furthermore, for patients with severe IRC, whether ICIs treatment should be discontinued, the NCCN guidelines recommend that for patients with severe IRC, the treatment with ICIs should be suspended and immunosuppressive therapy initiated. In this study, all patients with severe IRC had their ICIs treatment suspended. Although severe IRC usually requires a temporary suspension of ICIs, not all patients need to permanently discontinue treatment. In cases where ICIs were restarted after IRC remission, some patients did not experience IRC again, suggesting that after a careful individualized risk assessment, the use of ICIs can be resumed as appropriate. At present, there is no unified consensus on whether the timing of interruption of ICIs treatment will affect their anti-tumor efficacy. Theoretically, ICIs exert their anti-tumor effects by activating T cells, and this effect does not immediately disappear after drug withdrawal but gradually weakens over time. This view is also supported by clinical observations, where tumor volume continues to shrink or the disease remains stable after ICIs treatment is stopped. Therefore, we believe that a short interruption of ICIs treatment may not significantly affect the prognosis of patients, but if the interruption is too long, it may lead to a weakening of the anti-tumor effect.

This study found that patients with a history of UC are more prone to IRC. Therefore, it is of great significance to explore whether UC and IRC share certain underlying pathogenic mechanisms. In the diseased mucosa of UC patients, the proportion of helper T cells 17 (Th17) cells, which mainly secrete interleukin-17A (IL-17A), is significantly increased. As a pro-inflammatory factor, interleukin-6 (IL-6) promotes the differentiation of Th17 cells and induces the production of IL-17A, leading to intestinal mucosal damage and is considered a key link in the pathogenesis of UC. Previous studies have also shown that in the intestinal tissue samples of IRC patients, the levels of IL-6 and the proportion of Th17 cells are also elevated, which is consistent with our observations (25, 26). Therefore, we speculate that the IL-6-Th17 signaling pathway may play an important role in the pathogenesis of both UC and IRC. Additionally, we observed that in UC patients without IRC, the levels of IL-6 and IL-17A in their bodies were still slightly higher than those in the healthy patients, suggesting that the intestinal immune system of UC patients may be in a pre-activated state, which may explain why UC patients are more prone to IRC. Another T cell subtype - regulatory T (Treg) cells expressing PD-1/PD-L1, also plays a core role in maintaining intestinal immune homeostasis. Their compensatory upregulation is an important adaptive response in the chronic inflammatory microenvironment of UC (27). This upregulation essentially represents a negative feedback mechanism initiated by the host to suppress excessive immune activation. Notably, in the chronic inflammatory environment of UC patients, although the number of Tregs remains relatively stable, their function is significantly impaired, mainly manifested as downregulated CTLA-4 expression, reduced IL-10 secretion, and selective depletion of the Treg subset with high PD-1 expression. When the PD-1 pathway is blocked by inhibitors, the remaining Tregs will completely lose their immunosuppressive ability, leading to uncontrolled effector T cells and exacerbating the inflammatory response (28).

Furthermore, a study has shown that mice transplanted with the intestinal microbiota of UC patients had a significantly increased incidence of colitis after treatment with PD-1 inhibitors (29). This study, from the perspective of the interaction between the intestinal microbiota and the immune system, reveals the potential role of the intestinal microbiota in the co-pathogenesis of UC and IRC. Another report published in Nature pointed out that anti-TNF-α treatment can significantly improve IRC symptoms, and infliximab (a classic anti-TNF-α drug) is also one of the main drugs for treating UC at present (30). In summary, there are multiple commonalities in the pathogenesis and treatment strategies between UC and IRC, and these cross-factors may be an important reason why UC patients are more prone to IRC. Therefore, actively managing the underlying UC during immunotherapy and providing early intervention with IL-6 antibodies to patients with higher baseline IL-6 levels are of great significance for preventing severe IRC.

This study has several limitations. Firstly, due to the retrospective study design, there is a risk of selection bias and partial data loss. Secondly, although the sample size of this study is the largest in this field to date, it is still relatively limited, which restricts our further subgroup analysis. Thirdly, given that UC is usually an exclusion criterion in various immunotherapy clinical trials, the use of ICIs is entirely determined by the attending physician based on clinical judgment, lacking a unified medication standard. Fourth, there is currently no recognized diagnostic criterion to distinguish UC recurrence from IRC. Moreover, many advanced cancer patients are reluctant to undergo invasive procedures such as endoscopy or biopsy when experiencing intestinal symptoms. Therefore, accurately identifying IRC and differentiating it from other types of enteritis remains a significant challenge in clinical practice, a problem not only present in this study but also a common issue in this field. Fifth, this study involves multiple medical centers, and due to differences in diagnostic and treatment standards among institutions, especially in the absence of unified guidelines, it may have an impact on the study results. Sixth, patients with severe IRC often experience treatment interruption and the use of corticosteroids. Due to the limited sample size, we were unable to further evaluate and exclude the influence of these factors on prognosis. Seventh, this study included multiple anti-PD-1 antibodies. Different types of anti-PD-1 antibodies may introduce a certain degree of heterogeneity, which could potentially affect the research results. Due to the limited sample size, we were unable to conduct further subgroup analyses to explore their specific impacts.

## Conclusion

In summary, our findings suggest that the preexisting UC increases the risk of IRC in patients treated with ICIs. Notably, patients with mild IRC show better tumor control and longer survival, while the survival benefit for those with severe IRC is not obvious. Additionally, the serum levels of IL-6 and IL-17A in IRC patients are significantly elevated. Therefore, for preexisting UC patients treated with ICIs: mild IRC may suggest a favorable prognosis, and being vigilant and effectively managing the occurrence of severe IRC is crucial for maximizing clinical benefits. Targeting the IL-6 pathway may be a potential new strategy for treating IRC in the future.

## Data Availability

The raw data supporting the conclusions of this article will be made available by the authors, without undue reservation.
