# Supplementary material for: Preexisting ulcerative colitis increases the risk of immune-related colitis and predicts divergent survival outcomes in gastrointestinal cancer patients treated with immune checkpoint inhibitors
Source: Front Immunol. 2025 Aug 13;16:1627680. doi: 10.3389/fimmu.2025.1627680 (PMC12380752; doi:10.3389/fimmu.2025.1627680)
Supplement: Supplementary file 1 [file DataSheet1.docx]

Supplementary Figures

**
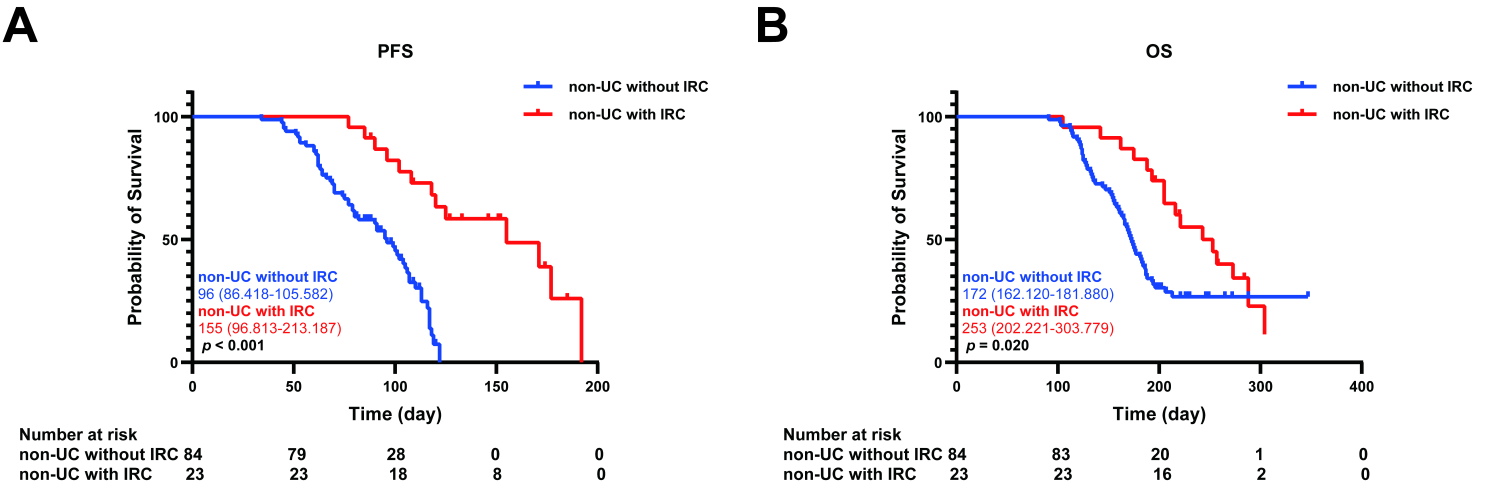
**

**Supplementary Figure 1.** Kaplan-Meier Survival Curve of PFS and OS. A: The Kaplan-Meier curve of PFS (non-UC without IRC vs non-UC with IRC). B: The Kaplan-Meier curve of OS (non-UC without IRC vs non-UC with IRC).
