# Supplementary material for: Preexisting ulcerative colitis increases the risk of immune-related colitis and predicts divergent survival outcomes in gastrointestinal cancer patients treated with immune checkpoint inhibitors
Source: Front Immunol. 2025 Aug 13;16:1627680. doi: 10.3389/fimmu.2025.1627680 (PMC12380752; doi:10.3389/fimmu.2025.1627680)
Supplement: Supplementary file 2 [file Table1.docx]

**Table S1** Treatment information of overall patients

| Type of cancer | Type of ICIs | No. of Patients |
| --- | --- | --- |
| ESCC | Tislelizumab | 2 |
|  | Pembrolizumab | 8 |
|  | Camrelizumab | 6 |
|  | Sintilimab | 8 |
| GC | Tislelizumab | 13 |
|  | Pembrolizumab | 28 |
|  | Camrelizumab | 19 |
|  | Sintilimab | 9 |
|  | Toripalimab | 1 |
| HCC | Sintilimab | 8 |
|  | Pembrolizumab | 12 |
|  | Camrelizumab | 8 |
|  | Tislelizumab | 12 |
| CRC (MSI-H) | Pembrolizumab | 4 |
