# Supplementary material for: Preexisting ulcerative colitis increases the risk of immune-related colitis and predicts divergent survival outcomes in gastrointestinal cancer patients treated with immune checkpoint inhibitors
Source: Front Immunol. 2025 Aug 13;16:1627680. doi: 10.3389/fimmu.2025.1627680 (PMC12380752; doi:10.3389/fimmu.2025.1627680)
Supplement: Supplementary file 3 [file Table2.docx]

**Table S2** Immunosuppressive therapy for UC

| Immunosuppressant | No. of Patients |
| --- | --- |
| Corticosteroids | 5 |
| Vedolizumab | 1 |
| Adalimumab | 1 |
| Azathioprine | 1 |
